# Pharmacodynamics of Oral Cholecalciferol in Healthy Individuals with Vitamin D Deficiency: A Randomized Open-Label Study

**DOI:** 10.3390/nu13072293

**Published:** 2021-07-02

**Authors:** Angelo Fassio, Davide Gatti, Maurizio Rossini, Camilla Benini, Elena Fracassi, Eugenia Bertoldo, Ombretta Viapiana, Stefano Milleri, Matteo Gatti, Giovanni Adami

**Affiliations:** 1 Rheumatology Unit, University of Verona, 37134 Verona, Italy; maurizio.rossini@univr.it (M.R.); camilla.benini@yahoo.it (C.B.); elena.fracassi@univr.it (E.F.); eugenia.bertoldo@gmail.com (E.B.); ombretta.viapiana@univr.it (O.V.); matgat93@gmail.com (M.G.); adami.g@yahoo.com (G.A.); 2 Centro Ricerche Cliniche di Verona, 37134 Verona, Italy; Stefano.Milleri@crc.vr.it

**Keywords:** vitamin D, cholecalciferol, osteoporosis, bone turnover markers, supplementation, pharmacodynamics

## Abstract

Comparative pharmacodynamic (PD) analyses on different dosing schedules for cholecalciferol supplementation are limited. This was an open-label, randomized, parallel-group study involving 75 healthy individuals deficient in vitamin D (baseline 25OHD < 20 ng/mL) receiving oral cholecalciferol with three different dosing regimens: Group A: 10,000 IU/day for 8 weeks followed by 1000 IU/day for 4 weeks; Group B: 50,000 IU/week for 12 weeks and Group C: 100,000 IU every other week for 12 weeks. Regulators of calcium and phosphate homeostasis, bone turnover markers and Wnt inhibitors were measured at baseline, Day 28, 53, 84, and 112. The 1,25OH2D increased at each time point. The increase was greater (*p* < 0.05) for group A vs. B and C at Day 28, and vs. group B at Day 56. No significant difference among groups was observed for the other biomarkers. The 24,25OH2D remained stable over time. PTH decreased at Day 84 and FGF-23 increased at all time points. CTX-I and PINP increased slightly at Day 28. BALP decreased from Day 56 onward. Dkk-1 increased from Day 56 onward, while sclerostin did not show significant changes. In healthy individuals deficient in vitamin D, vitamin D supplementation exerted effects on multiple regulators of calcium, phosphate and bone metabolism, without marked differences using the three regimens.

## 1. Introduction

Vitamin D is a fundamental compound for bone and mineral metabolism health [1], and it is involved in the regulation of calcium-phosphorus homeostasis and the maintenance of the musculoskeletal system [2].

In humans, vitamin D can be synthesized endogenously from 7-dehydrocholesterol in the skin by absorbing ultraviolet B (UVB) radiation. Alternatively, vitamin D can be obtained from the diet or dietary supplementation in the form of either vitamin D3 (cholecalciferol) or D2 (ergocalciferol).

Vitamin D requires a two-step activation process to become hormonally active. Vitamin D3, a compound with a half-life of approximately 24 h [3], is transported in the bloodstream bound to the vitamin D binding protein. Within hours, vitamin D is taken up following synthesis or dietary uptake, and activated mainly via the liver to 25-hydroxyvitamin D (25OHD), and then the kidney to 1α,25-dihydroxyvitamin D (1,25OH2D) [4] (calcitriol, a metabolite with a very short half-life, estimated to only be a few hours [3]). Often, the C24 oxidation pathway by CYP24A1 is thought to be the main removal pathway for 1,25OH2D and 25OHD, with their conversion in 1,24,25-trihydroxyvitamin D and 24R,25-dihydroxyvitamin D (24,25OH2D), respectively. Indeed, the vitamin D metabolome is much more complex, and different pathways with many other intermediates are involved, with many questions yet to be answered, especially concerning the regulation and the destiny of all these compounds [3].

To date, several epidemiological reports have shown an association between low vitamin D serum levels to several human diseases [5,6]. On the other hand, interventional studies on cholecalciferol supplementation for extra-skeletal benefits are still inconsistent [6] and often affected by design/methodological flaws [7].

It is currently recognized and well-documented that low and very low vitamin D levels (i.e., below 20 and 10 ng/mL respectively) are associated with impaired bone health [2]. Nevertheless, there is a significant lack of agreement on the most appropriate regimen for vitamin D supplementation (dosage, administration schedule, treatment duration, etc.) [2]. This heterogeneity can be partially explained by the scarcity of comparative pharmacokinetics (PK) and pharmacodynamic (PD) data for the different supplementation regimens [8,9,10]. Interestingly, in this regard, there is growing evidence suggesting that the treatment schedule itself (i.e., boluses vs. frequent administrations) may impact differently on the effectiveness of the treatment [11,12,13] and on clinical outcomes. Several studies and a few meta-analyses seem to suggest more promising results with frequent administration schedules on skeletal and extra-skeletal outcomes [6,7,14,15,16,17,18]. Recently, we published data comparing the PK profile of three different cholecalciferol supplementation schedules (Group A: 10,000 IU/day for eight weeks followed by 1000 IU/day for four weeks; Group B: 50,000 IU/week for 12 weeks, Group C: 100,000 IU/every other week for 12 weeks) and normalization of 25 (OH)D serum levels was quickly achieved using all three dosing regimens [19]. In the present study, we describe the results of this study focusing on the PD effects of these three different treatment regimens on vitamin D metabolites, regulators of calcium and phosphate homeostasis and bone turnover markers (BTMs), and Wnt inhibitors.

## 2. Materials and Methods

### 2.1. Patients and Study Design

This was a single-centre, open-label, randomized, parallel group phase I (bioequivalence) study in male and female healthy individuals that was aimed to specifically compare the PK and PD profiles of cholecalciferol (DIBASE^®^, Abiogen Pharma, Pisa, Italy) administered as repeated once daily (10,000 IU/day for 8 weeks followed by 1000 IU daily for 4 weeks; group A), weekly (50,000 IU/week for 12 weeks; group B), and in alternate week doses (100,000 IU every other week for 12 weeks; group C). The regimens adopted in this trial correspond to the highest dosages allowed for oral cholecalciferol (DIBASE^®^) in Italy, according to its Summary of Product Characteristics (SmPC), for the correction of vitamin D deficiency in adults [20]. This study was undertaken (first enrollment to end of study) from September 2017 to June 2018.

Inclusion criteria were: Caucasian males and females aged 18–60 years, with body mass index (BMI) from 18.5 kg/m^2^ to 28 kg/m^2^; 25OHD value <20 ng/mL and negative for urine pregnancy test. Exclusion criteria were: a history of alcohol or drug abuse, drinking excessive amounts of tea, cocoa, coffee and/or beverages containing caffeine (>5 cups/day) or wine (>0.5 L/day) or spirits (>50 mL/day) on a regular basis, abnormal diets (<1600 or >3500 kcal/diet) or substantial changes in eating habits within the past 4 weeks, use of any medicines including antibacterial drugs, over-the-counter medication, vitamins, and natural products in the previous 2 weeks; a history of clinically significant gastrointestinal, renal (including renal stone formation), liver, pulmonary, endocrine, oncologic, or cardiovascular disease; or history of epilepsy, asthma, diabetes mellitus, psychosis, or severe head injury; vitamin D therapy or food supplements taken within the past two months; metabolic disorders of calcium or bones (including secondary hyperparathyroidism), history of angina pectoris, or artificial UVB exposure (solarium) in the previous 14 days.

Approval for this study was obtained by the Institutional Research Committee (protocol identification: DIBA/11, EudraCT Number: 2017-000194-36) in accordance with the 1964 Helsinki declaration. Written informed consent was obtained from all participants included in the study. The primary objective of this study was to compare the pharmacokinetic profiles of cholecalciferol (DIBASE^®^) and calcium, phosphate, and albumin changes when administered as repeated once daily, weekly, and in alternate weekly doses in healthy male and female individuals. The full study protocol and the results related to the primary outcome have already been published elsewhere [19].

### 2.2. Laboratory Analysis

The secondary objective of this study was to perform an exploratory analysis on the PD profiles in serum levels of 1,25-dihydroxyvitamin D (1,25OH2D), 24,25-dihydroxyvitamin D (24,25OH2D), parathyroid hormone (PTH), ionized calcium, fibroblast growth factor-23 (FGF-23), C-terminal telopeptide of type I collagen (CTX-I), procollagen type I N-terminal propeptide (PINP), bone alkaline phosphatase (BALP), Dicckopf-1 (Dkk-1), and sclerostin.

After screening, eligible patients were randomized in a 1:1:1 ratio using a randomization list generated with computer software. In all groups A, B, and C, blood samples for PD analysis were collected pre-dose on each of on Days 1, 28, 56, 84, and 112.

These samples were stored at −70 °C until the end of the study, at which point all of them were assayed for the following BTMs: PINP (IDS-iSYS Intact PINP, IS-4000), CTX-I (IDS-iSYS CTX-I CrossLaps, IS-3000), PTH (IDS-iSYS Intact PTH, IS-3200), BALP (IDS-iSYS Ostase BAP, IS-2800), 24,25OH2D (Human (24R) 24,25 (OH)2 Vit. D3, MyBioSource, MBS109076), Dkk-1 (DKK1, Biomedica, BI-20413), Sclerostin (SOST, Biomedica, BI-20492), and FGF-23 (FGF23 C-terminal, Biomedica, BI-20702). Serum PINP, CTX-I, PTH, and BAP were measured by the IDS-ISYS Multi-Discipline automated analyzer (Immunodiagnostic System, Boldon, UK) based on chemiluminescence technology. Serum 24,25OH2D, Dkk-1, sclerostin, and FGF-23 were measured by ELISA immunoassays (MyBioSource San Diego, CA, Biomedica Medizinprodukte, Wien, Austria, and Diaclone SAS, F-25020 Besancon Cedex, France) on the Fully Automated Microplate Analyser Personal LAB (Adaltis Italia). The overall intra-assay coefficient of variation (CV) and inter-assay CV were, respectively: PINP (2.87% and 4.63%), CTX-I (3.22% and 6.16%), PTH (2.7% and 5.5%), BAP (1.56 and 7.28%), 24,25OH2D (both < 15%), Dkk-1 (both ≤ 3%), Sclerostin (≤7% and ≤10%), and FGF-23 (≤12% and ≤10%). The 1,25OH2D was analyzed with 1,25OH2D Vit.D XP (IDS-iSYS 1,25(OH)2 Vit.D XP IS-2000). The intra-assay CV was 7.57% and the inter-assay CV was 10.85%.

### 2.3. Statistical Analysis

Statistical analysis was performed by Advice Pharma Group S.r.l., Milano, Italy using SPSS software, Version 22 (SPSS, Inc., Chicago, IL, USA). Analysis of variance (ANOVA) followed by post-hoc analysis (Bonferroni) and a two-sided Student’s *t* test were used to estimate the absolute differences between groups (A vs. B vs. C). Two-sided *p* values of 0.05 or lower were considered statistically significant. Data are presented as mean ± SD. For continuous variables, the number of non-missing values (N), mean and standard deviation (SD), and median and interquartile range (IQR) are presented. To test differences between groups of treatment in terms of change in PD parameters over time, the analysis of variance (ANOVA) and post hoc tests (not corrected and corrected with the Holm–Bonferroni step-down correction) were computed. In order to evaluate for a possible influence of baseline BMI on the tested biomarkers (in the overall cohort and among each treatment subgroup), we tested the differences at baseline of all the biomarkers between individuals with BMI > and ≤22.82 Kg/M^2^ (median BMI value of the overall cohort) through Student’s *t*-test. A similar analysis was performed for the absolute changes from baseline to day 28, 56, 84, and 112 for the overall cohort and in each treatment subgroup for all the investigated biomarkers. The relationship between two variables at a specific time point were assessed using the Pearson correlation coefficient (r). This study did not include a formal power calculation. The sample size of 25 participants per arm was primarily based on practical considerations. However, 25 individuals per group would allow for the detection of a change in trough 25(OH)D concentration from a baseline of 16 ng/mL with a statistical power of 80% and a Type I error of 0.05 and an 80% probability of detecting an adverse event (AE) with an underlying incidence rate of 0.07 [19].

## 3. Results

### 3.1. Baseline Characteristics

**Figure 1:**
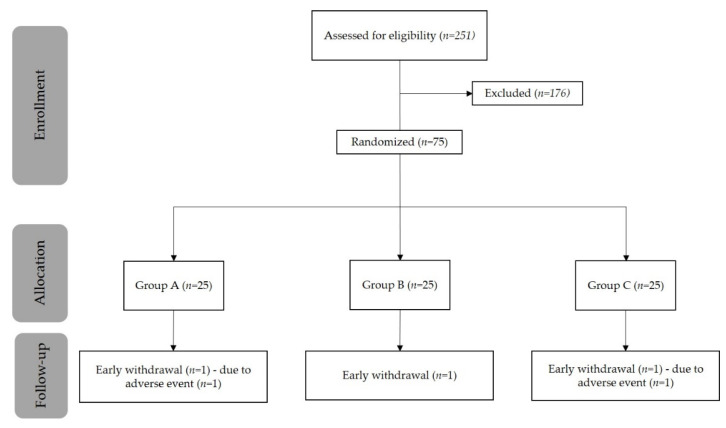
Patient disposition.

In total, 251 healthy volunteers were screened for eligibility (Figure 1). Of these, 75 participants were randomized to treatment (25 in each treatment arm), 73 volunteers completed the study, and 2 prematurely discontinued. One subject in Treatment A discontinued due to an adverse event (reported as skin rash with mild severity and not related to the treatment), and one subject in Treatment C discontinued due to withdrawal of consent. Compliance to treatment was 100% for each group at each evaluation.

Data on total serum calcium, phosphate, and albumin and on the differences in 25OHD exposures have already been previously published [19]. Demographic and biochemical characteristics at baseline are presented in Table 1 and Table 2, respectively.

No statistically significant differences were found among the three groups in terms of age, weight, and BMI (Table 1 and Supplementary Table S1). Examining baseline differences according to BMI (BMI > vs. ≤ of the median value), we did not find a statistically significant difference for any of the vitamin D metabolites (25OHD, 1,25OH2D or 24,25OHD2), but we did observe a difference in Dkk-1 (22.33 ± 11.36 vs. 14.95 ± 9.01 pmol/L, *p* =0.003 for BMI >22.82 and ≤22.82, respectively) and in FGF-23 (1.41 ± 2.08 vs. 0.54 ± 0.44 pmol/L, *p* = 0.15).

When we compared the absolute changes from baseline of the overall cohort between individuals with BMI > and ≤22.82 Kg/M^2^ we did not observe a statistically significant difference between the two subgroups for any of the vitamin D metabolites. The only significant difference found was for the changes in Dkk-1 serum level from baseline to day 28 (−1.00 ± 5.53 vs. 1.99 ± 5.03 pmol/L, *p* = 0.018 for BMI >22.82 and ≤22.82, respectively), day 56 (0.72 ± 8.12 vs. 7.21 ± 7.86 pmol/L, *p* = 0.001), and day 84 (3.0 ± 6.84 vs. 9.15 ± 10.8, *p* = 0.006). When we analysed the three different treatment subgroups separately, no significant difference according to BMI was found in group A and B, while in subgroup C it remained significant at day 56 (−0.23 ± 8.78 vs. 10.86 ± 8.6 pmol/L, *p* = 0.007 for BMI >22.82 and ≤22.82, respectively) and day 84 (0.84 ± 4.37 vs. 10.54 vs. 13.13 pmol/L, *p* = 0.018). Finally, we found a weak but positive correlation between baseline BMI values and the magnitude of the changes in serum Dkk-1 over time in the overall cohort (r = 0.22, *p* = 0.001).

### 3.2. Laboratory Parameters

**Figure 2:**
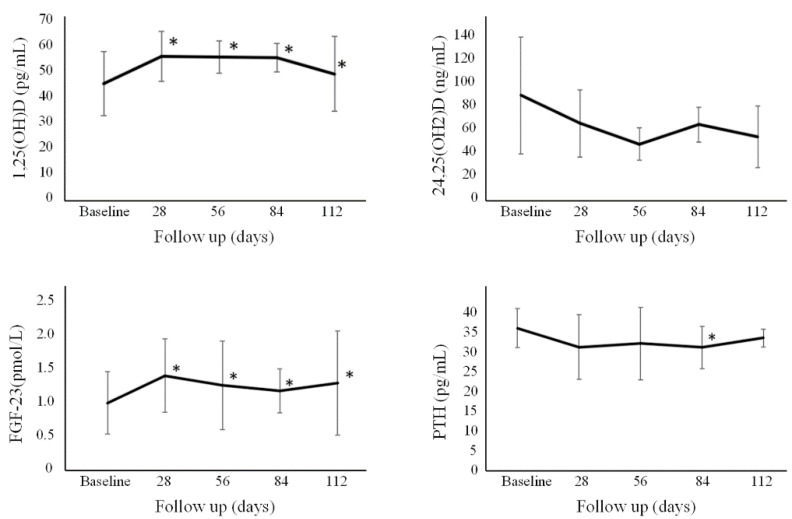
Absolute changes of the overall cohort of 1,25OH2D, 24,25OH2D, PTH, and FGF-23. * *p* < 0.05 with respect to baseline. Error bars denote standard deviation. FGF-23 = fibroblast growth factor-23; PTH = parathyroid hormone.

**Figure 3:**
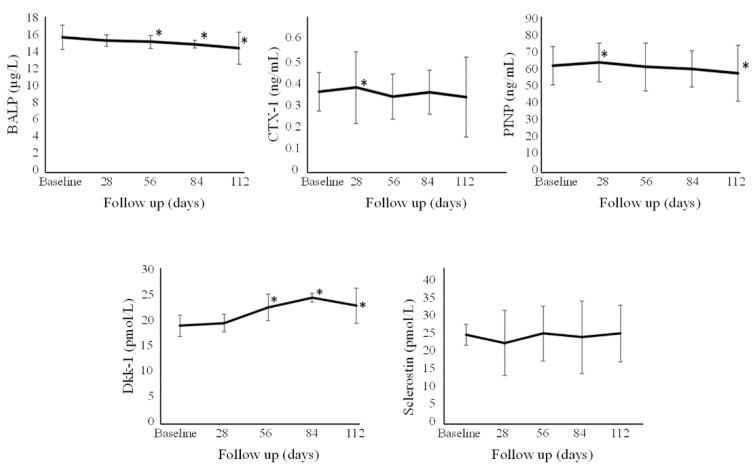
Absolute changes of the overall cohort of BALP, CTX-I, PINP, Dkk-1, and sclerostin. * *p* < 0.05 with respect to baseline. Error bars denote standard deviation. BALP = bone alkaline phosphatase; CTX-I = C-terminal telopeptide of type I collagen; DKK-1 = Dicckopf-1; PINP = procollagen type I N-terminal propeptide.

The absolute values of the tested biochemical markers over time are reported in Figure 2 and Figure 3 and Supplementary Table S2. Percentage changes with respect to baseline of the various biochemical parameters in the single Group are reported in Supplementary Table S3.

No statistically significant difference was observed at baseline between Group A, Group B, and Group C for the PD parameters examined, except for CTX-I (higher in Group A) and PINP (higher in Group A).

Overall levels of 1,25-dihydroxyvitamin D for all individuals showed an increase at Day 28, Day 56, Day 84, and Day 112. This increase was greater (*p* < 0.05) for Group A vs. B and C at Day 28, and vs. just group B at Day 56. Ionized calcium for all individuals slightly decreased after one month and at Day 56 and 84, with no significant differences among the different groups. Overall, no significant change was found for 24,25-dihydroxyvitamin D over time. PTH significantly decreased after three months. FGF-23 levels for all individuals were observed to increase at all time points, with no significant differences among the different groups. CTX-I levels showed a slight increase in all individuals at the first month at Day 28 only, with no significant differences among the different groups. P1NP levels were observed to significantly increase in all individuals at Day 28 but then significantly decreased at Day 112, with no significant differences among the different groups. BALP levels in all individuals slightly decreased from Day 56 onwards, with no statistically significant differences among the different groups. There was a statistically significant increase (*p* < 0.05) in the level of Dkk-1 for Groups A, B, and C at Day 56, 84, and 112 compared to baseline levels. Sclerostin remained stable over time, without significant differences seen among the different groups.

When the absolute changes from baseline to week 4, week 8, and week 12 were correlated (all groups together), we found that: 1,25OH2D showed a very weak positive correlation with Dkk-1 (r = 0.15, *p* = 0.024), 24,25OH2D had a very weak negative correlation with PTH (r = −0.14, *p* = 0.038), PTH had a weak positive correlation with CTX-I (r = 0.325, *p* < 0.001), and finally, BALP showed a weak positive correlation with PINP (r = 0.46, *p* < 0.001). Furthermore, when the present data were correlated with those of 25OHD serum levels, we observed a very weak positive correlation between the changes in 25OHD and 1,25OH2D (r = 0.18, *p* = 0.009), with Dkk-1 (r = 0.17, *p* = 0.013) and a very weak negative correlation with the changes in CTX-I (r = −0.13, *p* = 0.048).

## 4. Discussion

In this study, we described the PD of cholecalciferol supplementation on healthy individuals deficient in vitamin D and compared the effects of three different administration schedules. Differences in PK among the three different groups have already been reported in a previous paper [19], and showed a higher systemic 25OHD exposure in the group treated with the daily dose. Conversely, our present data demonstrate noteworthy effects on PD parameters, but do not show any significant differences in the effect of the administration schedule on vitamin D metabolites (24,25OH2D and 1,25OH2D), endocrine and autocrine/paracrine bone-regulating mediators (PTH, FGF-23 and Dkk-1 and sclerostin, respectively), and bone turnover markers (CTX-I, PINP, and BALP).

When considering the changes observed over time, as expected, we found an increase in 1,25OH2D (+31–34%), slightly more pronounced early in the group treated with the daily dose, arguably due to a greater systemic 25OHD exposure [19]. This was associated with a very mild increase in serum calcium and phosphate, but no case of hypercalcemia was detected, as reported in our previous paper [19]. Here we observed that the levels of ionized calcium changed slightly and in opposite directions, opposing the possibility of a clinically worrisome influence on blood calcium. The increase in calcium and phosphate was also accompanied by a decrease in PTH and an increase in FGF-23 serum levels, consistent with a homeostatic response. While the relationship between vitamin D supplementation and serum PTH is well-known, the one between vitamin D and FDG-23 has been a very recent topic of interest. Indeed, our results are in line with data from a recent meta-analysis documenting an associated increase in serum FGF-23 with cholecalciferol supplementation [21]. The increase was especially seen when the levels of serum 25OHD exceeded 100 nmol/L and starting with baseline levels <50 nmol/L [21], explained by the vitamin D-induced increase in phosphorus absorption, which reaches its plateau once adequate 25OHD concentrations (i.e., ≥50 nmol/L) are achieved [21].

Interestingly, we did not observe any significant change in the levels of 24,25OH2D, a metabolite produced by the action of CYP2A1 [3]. This compound represents the first metabolite in the removal of 25OHD of the C24-oxydation pathway [3]. In the past, 24,25OH2D was thought to be an inert catabolic product of vitamin D, but more recent evidence supports the possibility of an inherent biological activity [22,23,24]. In addition, 24,25OH2D is considered to reflect vitamin D receptor’s (VDR) activity (under the influence of 1,25OH2D) [3,24]. Our study enrolled young and healthy participants deficient in vitamin D without any evident clinical or biochemical signs of osteometabolic distress (i.e., secondary hyperparathyroidism). In our opinion, in this healthy cohort devoid of any actual endocrine impairment of the PTH-1,25OH2D axis, the administration of cholecalciferol was not associated with a significant effect on VDR activity. Indeed, previous studies have shown a strong correlation between the serum 24,25OH2D and 25OHD [3]. However, many of these studies involved individuals burdened with significant risk factors or conditions known to affect bone metabolism, such as multiple sclerosis [25], pregnancy/lactation [26], or patients with overt osteomalacia who received treatment with vitamin D [27]. Therefore, in our setting, it is intriguing to speculate on the destiny of the supplemented cholecalciferol. To explain the lack of a significant change in 24,25OH2D, we speculate that, in our healthy cohort, most of the administered cholecalciferol was removed through other pathways, and converted in one or more of the many non-VDR interacting metabolites currently known [3].

Previous studies on a single high-dose cholecalciferol bolus (i.e., 600,000 IU) showed a marked increase (+50%) in serum CTX-I and P1NP, persisting for over 2 months [28,29]. Conversely, in individuals receiving smaller boluses (300,000 IU or 100,000 IU), only a mild and temporary increase was observed [28]. Indeed, this subacute effect in bone resorption has been hypothesized to contribute to the lack of benefit observed in some studies (or even the unexpected increase in fracture rate) reported shortly after the administration of a high dose of vitamin D3 [30]. Our present data emphasize the importance of the treatment schedule in the correction of hypovitaminosis D; indeed, despite administering a total cumulative dose identical to the single megadose in the study by Rossini et al. [28], we did not observe any notable short-term bone effects on bone turnover markers in our cohort. The increase we observed in CTX-I, and PINP, was modest (+12% and +4.9%, respectively) and only temporarily seen, at Day 28. The regimens adopted in this trial correspond to the highest dosages allowed for cholecalciferol (DIBASE^®^) in Italy, according to its SmPC, for the correction of vitamin D deficiency in adults [20]. Given their effects, especially on markers of bone resorption, larger doses of cholecalciferol for the correction of vitamin D deficiency may not be advisable.

PINP serum levels at Day 112 showed a statistically significant decrease when compared to baseline (−7.1%). Interestingly, we noted a trend towards a stable decrease for BALP, that was mild (<10%) but consistently statistically significant. Usually, in conditions such as symptomatic vitamin D deficiency or overt osteomalacia, after adequate vitamin D supplementation, a decrease of BALP serum level with a return to baseline is seen, as known since the seminal study by Papapoulos et al. [27]. In our cohort, as already mentioned, no clinical or biochemical suggestion of endocrine impairment was discernible, with serum levels of baseline PTH in the mid-range of the reference interval. However, to interpret the decrease in BALP after cholecalciferol supplementation, the presence of a very mild BALP hypersecretion associated with the deficiency status might be hypothesized. This may involve a mild increase in the osteoblast activity needed to compensate for a relative decrease in the availability of calcium and phosphate for mineralization. In this way, the vitamin D repletion might explain the subsequent decrease in blood concentrations of this enzyme.

Data on the effect of vitamin D supplementation on Wnt pathway inhibitors (Dkk-1 and sclerostin) are currently lacking. Our data showed a stable increase in Dkk-1 serum levels from Day 56 onwards, without any significant change in serum sclerostin. There is in vitro evidence suggesting the induction of Dkk-1 gene transcription by 1,25OH2D in cancer cells [31,32] and in osteoblasts [33]. In addition, while there is laboratory evidence suggesting the upregulation of sclerostin by 1,25OH2D, data on clinical studies are not always in line with this [34,35,36,37,38,39]. Our data failed to show any detectable change in this marker; these inconsistencies are, to date, difficult to explain and are probably due to the different diseases where these Wnt inhibitors were explored. On one hand, Dkk-1 has been found to be dysregulated (excessively expressed) in rheumatic disease characterised by systemic inflammation with local/systemic bone loss [40,41] and to decrease after successful anti-inflammatory treatment [42,43,44], with sclerostin being excessively elevated in patients deficient in vitamin D with chronic kidney disease and associated with poor fracture outcomes [45]. On the other hand, both have been shown to decrease or to increase (arguably as a homeostatic response) after anti-resorptive and anabolic treatment for osteoporosis [46,47,48]. We also observed a possible link between the magnitude of its changes and individuals’ BMI, with the observation of more pronounced increases in those with a higher BMI. However, based on these premises, it is currently challenging to determine whether the observed increase in Dkk-1 was a direct effect on its expression (as some mechanistic data would suggest) or a counter-regulative response to preserve a healthy bone homeostasis.

## 5. Study Limitations

Our study has some limitations worth mentioning. Participants received oral cholecalciferol supplementation for only 12 weeks. A longer treatment period may have revealed additional information on the endpoints examined. As reported previously in detail [19], treatment at the maximum allowed dose of 10,000 IU can result in mild or moderate side effects that are important to monitor. We measured the vitamin D metabolites’ serum level by immunoenzymatic reaction (ELISA), an assay that may be affected by different variables (i.e., pregnancy, severe illnesses) [49,50]. Nevertheless, we studied a healthy (although vitamin D deficient) population, and for this reason we do not expect these issues to undermine our results. For the same reason, caution should be taken before generalizing our results to a diseased population (i.e., the frail elderly, patients affected by chronic illnesses, malabsorption), because, as already discussed, the absorption and/or the destiny of vitamin D metabolites might be different in these settings.

One final limitation is the absence of data specifically regarding the percentage of fat mass in enrolled individuals, as excess adiposity may influence vitamin D metabolism [51]. However, given the fact that this was a young healthy population with little variation in BMI values outside the normal range (no patients were actually obese) and also absent for comorbid diseases, we do not believe that the absence of data on fat mass poses a concern with regard to the interpretation of our results.

Furthermore, we studied the changes in 25OHD, 1,25OH2D, and 24,25OH2D after cholecalciferol administration, but the vitamin D metabolome is extremely complex and articulated, with dozens of compounds that can originate from vitamin D3 (or D2) [3]. Exactly 100 years after the discovery of vitamin D [52], we still ignore many significant aspects of its metabolism and are unable to explain why different treatment schedules might be associated with different clinical outcomes, especially when dealing with extra-skeletal effects [6,7,14,15,16,17,18].

## 6. Conclusions

In conclusion, the results of our study show important effects on multiple regulators of calcium, phosphate, and bone metabolism of vitamin D supplementation in healthy individuals deficient in vitamin D, without any major differences among the three treatment schedules. Future studies should further investigate the complex vitamin D metabolome after cholecalciferol administration, and also involve more fragile patients with biochemical/clinical evidence of impaired bone metabolism.

## Figures and Tables

**Table 1:** Baseline demographic characteristics.

Baseline Characteristics		Total Population (*n* = 75)	Group A (*n* = 25)	Group B (*n* = 25)	Group C (*n* = 25)	*p*-Value (ANOVA)
Sex						
Male	N (%)	31 (41.3)	12 (48)	7 (28)	12 (48)	NS
Female	N (%)	44 (58.7)	13 (52)	18 (72)	13 (52)	NS
Age (years)	Years (SD)	34.1 (10.2)	30.2 (10)	36.7 (8.8)	35.4 (11)	NS NS
Male	Years (SD)	33.4 (8.1)	31.1 (8.6)	34.6 (5.7)	35 (8.7)	NS NS
Female	Years (SD)	34.6 (11.6)	29.5 (11.4)	37.5 (9.7)	35.9 (13.1)	NS NS
Weight (Kg)	Mean (SD)	66.7 (12.4)	65.2 (13.5)	67.4 (9.8)	67.6 (13.7)	NS
Male	Mean (SD)	70.5 (12.2)	70.2 (10.4)	70.2 (13.3)	70.9 (14.3)	NS
Female	Mean (SD)	64.3 (12.2)	61.9 (14.7)	66.9 (10)	63 (12.7)	NS
BMI (Kg/M^2^)	Mean (SD)	23.1 (2.6)	22.6 (2.9)	23.4 (2.1)	23.2 (2.8)	NS
Male	Mean (SD)	23.7 (2.9)	23.4 (2.5)	24.7 (2.9)	23.3 (3.4)	NS
Female	Mean (SD)	22.6 (2.4)	21.7 (2.9)	23.5 (1.9)	22.4 (2.2)	NS

ANOVA = analysis of variance; BMI = body mass index; NS = not statistically significant: SD = standard deviation.

**Table 2:** Biochemical characteristics at baseline.

Time		Total Population (*n* = 73)	Group A (*n* = 24)	Group B (*n* = 25)	Group C (*n* = 24)	ANOVA *p*-Value
1,25-dihydroxyvitamin D (pg/mL)						
Baseline	N	73	24	25	24	0.23
	Mean (SD)	44 (11.5)	42 (8.8)	47.2 (12.7)	42.8 (12.3)	
	Median (IQR)	43.7 (36.5–50.2)	43.5 (36.5–49.2)	45.4 (38.2–54.8)	40.5 (35.9–48.1)	
		16.8–77.1	24.4–58.4	28.2–77.1	16.8–65.5	
24,25-dihydroxyvitamin D (ng/mL)						
Baseline	N	73	24	25	24	0.87
	Mean (SD)	93.9 (185.1)	98 (169)	105.6 (257.2)	77.7 (95.9)	
	Median (IQR)	33.2 (16–90.1)	31.5 (17.5–96.1)	29.4 (12.1–89.5)	39.7 (17.2–84.7)	
		4.9–1308	9.9–817.6	4.9–1308	5.7–361.6	
PTH (pg/mL)						
Baseline	N	73	24	25	24	0.32
	Mean (SD)	36.4 (14)	37.2 (15.4)	33.1 (10.5)	39.1 (15.7)	
	Median (IQR)	33.4 (25.9–43.1)	32.8 (25.6–43.1)	32.8 (25.5–40.9)	36 (27.2–48)	
		11.5–79.3	14.9–74.4	11.5–59.2	12.8–79.3	
Ionized calcium (mmol/L)						
Baseline	N	73	24	25	24	0.21
	Mean (SD)	1.2 (0)	1.2 (0)	1.2 (0)	1.2 (0)	
	Median (IQR)	1.2 (1.2–1.2)	1.2 (1.2–1.3)	1.2 (1.2–1.2)	1.2 (1.2–1.2)	
		1.2–1.3	1.2–1.3	1.2–1.3	1.2–1.3	
FGF-23 (pmol/L)						
Baseline	N	73	24	25	24	0.19
	Mean (SD)	1 (1.6)	0.5 (0.4)	1.3 (2.2)	1.1 (1.4)	
	Median (IQR)	0.5 (0.3–1)	0.4 (0.2–0.8)	0.6 (0.3–1.1)	0.6 (0.3–1.1)	
		0–10.8	0–1.6	0–10.8	0–5.4	
CTX-I (ng/mL)						
Baseline	N	73	24	25	24	0.02
	Mean (SD)	0.4 (0.2)	0.4 (0.2)	0.3 (0.2)	0.3 (0.1)	
	Median (IQR)	0.3 (0.2–0.5)	0.4 (0.3–0.5)	0.3 (0.2–0.4)	0.4 (0.3–0.4)	
		0.1–0.9	0.1–0.9	0.1–0.8	0.1–0.5	
P1NP (ng/mL)						
Baseline	N	73	24	25	24	0.003
	Mean (SD)	62.6 (24.1)	75.9 (29)	53.8 (18.7)	58.6 (18.2)	
	Median (IQR)	58 (45.9–72.2)	68.2 (57.9–90.9)	50.5 (42.5–57.1)	58.3 (43.5–71.4)	
		22.7–149.8	22.7–149.8	28.6–128.1	32.9–88.9	
BALP (ug/L)						
Baseline	N	73	24	25	24	0.08
	Mean (SD)	15.6 (6.7)	18 (8.5)	13.8 (3.9)	15.2 (6.6)	
	Median (IQR)	13.9 (11.1–18.3)	16.4 (12.5–20.8)	12.8 (11.1–15.6)	14.2 (10.4–18.3)	
		5.8–40.7	7.3–40.7	7.5–24.7	5.8–33.5	
Dkk-1 (pmol/L)						
Baseline	N	73	24	25	24	0.34
	Mean (SD)	18.6 (11.1)	16.7 (8.3)	17.7 (12.6)	21.2 (11.9)	
	Median (IQR)	15.7 (10.6–23.1)	17.3 (11.9–23.2)	13.7 (10.3–21)	18.6 (13.3–26.4)	
		2.2–56	2.2–32.6	4–50.3	6.1–56	
Sclerostin (pmol/L)						
Baseline	N	73	24	25	24	0.56
	Mean (SD)	25 (25.5)	20.5 (13.7)	28.2 (27)	26.1 (32.5)	
	Median (IQR)	18.9 (11.9–31.7)	16.8 (11.8–24.4)	19.5 (11.9–36.4)	19.2 (15.5–25.1)	
		4.1–173	4.1–66.2	5.8–139.1	5–173	

Comparisons were performed between groups (A vs. B vs. C) by analysis of variance; ANOVA; BALP = bone alkaline phosphatase; CTX-1 = C-terminal telopeptide of type I collagen; DKK-1 = Dicckopf-1; FGF-23 = fibroblast growth factor-23; IQR = interquartile range; P1NP = procollagen type I N-terminal propeptide; PTH = parathyroid hormone; SD = standard deviation.

## Data Availability

Data can be made available from the corresponding author upon request.

## Supplementary Materials

The following are available online at https://www.mdpi.com/article/10.3390/nu13072293/s1, Table S1: BMI median, interquartile range (IQR) and minimum and maximum values of the enrolled individuals at baseline, Table S2: Absolute levels of the biochemical parameters at baseline and at the various time points and Table S3: Percentage changes of the tested biochemical parameters with respect to baseline at the various time points.
