# Supplementary material for: Pharmacodynamics of Oral Cholecalciferol in Healthy Individuals with Vitamin D Deficiency: A Randomized Open-Label Study
Source: Nutrients. 2021 Jul 2;13(7):2293. doi: 10.3390/nu13072293 (PMC8308331; doi:10.3390/nu13072293)
Supplement: Supplementary file 1 [file nutrients-13-02293-s001.zip › nutrients-1265184-supplementary.pdf]

Supplementary Material Tables 1-3.

Supplementary Material Table 3. BMI median, interquartile range (IQR) and minimum and maximum values of the enrolled individuals at baseline.

Baseline		Total population (n=75)	Group A (n=25)	Group B (n=25)	Group C (n=25)
BMI (Kg/M^2)	Median [IQR]<br>Min-max	22.82<br>21.22-24.6<br>19-28	23.01<br>21.49-26.02<br>19-28	22.78<br>21.84-24.79<br>20-27	21.67<br>20.37-24.37<br>19-28
Males	Median [IQR]<br>Min-max	23.51<br>21.2-26.24<br>19-28	23.7<br>22.9-27.77<br>20-28	23.59<br>21.8-25.88<br>20-27	21.3<br>18.67-23.67<br>19-28
Females	Median [IQR]<br>Min-max	22.35<br>20.95-24.29<br>19-27	22.59<br>20.10-23.82<br>19-27	22.43<br>21.78-24.38<br>20-27	22.04<br>20.59-25.17<br>19-27

Supplementary Material Table 2. Absolute levels of biochemical parameters at baseline and at the various time points.

Time		ALL subjects (n=73)	Group A (n=24)	Group B (n=25)	Group C (n=24)	ANOVA p-value
**1,25-dihydroxyvitamin D (pg/mL)**						
Baseline	N<br>Mean (SD)<br>Median (IQR)	73<br>44 (11.5)<br>43.7 (36.5-50.2)<br>16.8-77.1	24<br>42 (8.8)<br>43.5 (36.5-49.2)<br>24.4-58.4	25<br>47.2 (12.7)<br>45.4 (38.2-54.8)<br>28.2-77.1	24<br>42.8 (12.3)<br>40.5 (35.9-48.1)<br>16.8-65.5	0.234
Day 28	N<br>Mean (SD)<br>Median (IQR)	73<br>57 (15.4)<br>56.6 (44.3-63.3)<br>33.8-110	24<br>61.6 (16.9)<br>59.9 (48.5-71.7)<br>37.4-110	25<br>57.5 (14.6)<br>57.3 (45.9-61.7)<br>36.1-95.2	24<br>52 (13.5)<br>50 (42.4-60)<br>33.8-85.7	0.091
Day 56	N<br>Mean (SD)<br>Median (IQR)	73<br>56.8 (11.7)<br>54.5 (50.6-63.7)<br>23.7-97	24<br>59.2 (9.3)<br>57.2 (52.8-64.4)<br>44.4-81.4	25<br>56.6 (12.1)<br>53.8 (49-57.7)<br>42.6-97	24<br>54.5 (13.2)<br>53.5 (46.2-63.3)<br>23.7-79.7	0.387
Day 84	N<br>Mean (SD)<br>Median (IQR)	72<br>56.3 (13.1)<br>55.4 (47-65.2)<br>28.3-96.6	24<br>55.8 (13)<br>53.5 (46.8-64.7)<br>32.1-83.9	24<br>57.5 (14.4)<br>56.5 (47-64.9)<br>28.3-96.6	24<br>55.6 (12.1)<br>53.5 (46.7-66.7)<br>37.4-74.9	0.859
Day 112	N<br>Mean (SD)<br>Median (IQR)	69<br>48.2 (13.4)<br>46.2 (39-55)<br>24-104	24<br>47.2 (11.1)<br>47.7 (41.2-55.2)<br>24-63.4	23<br>51.6 (17.2)<br>48.4 (39.7-62.4)<br>25.2-104	22<br>45.7 (10.7)<br>44.1 (39.2-48.5)<br>25.3-70.3	0.314
**24,25-dihydroxyvitamin D (ng/mL)**						
Baseline	N<br>Mean (SD)	73<br>93.9 (185.1)	24<br>98 (169)	25<br>105.6 (257.2)	24<br>77.7 (95.9)	0.866

	Median<br>(IQR)	33.2 (16-90.1)<br>4.9-1308	31.5 (17.5-96.1)<br>9.9-817.6	29.4 (12.1-89.5)<br>4.9-1308	39.7 (17.2-84.7)<br>5.7-361.6	
Day 28	N<br>Mean (SD)<br>Median<br>(IQR)	73<br>68.7 (152.8)<br>24.6 (16.8-53.5)<br>5.4-1077	24<br>77.4 (153.7)<br>24.4 (17.8-54.3)<br>8-760.7	25<br>80.4 (211.1)<br>24.6 (15.8-50.7)<br>5.5-1077	24<br>47.8 (48.4)<br>25 (18.9-63.8)<br>5.4-168.7	0.72
Day 56	N<br>Mean (SD)<br>Median<br>(IQR)	73<br>50.5 (76.1)<br>28.5 (11.2-59.8)<br>0-574.2	24<br>72.4 (115.4)<br>37.8 (15.7-77.3)<br>6.6-574.2	25<br>43 (50.6)<br>23.1 (10.6-50)<br>0-184.4	24<br>36.6 (36.8)<br>22 (10.3-51)<br>7.5-133.5	0.22
Day 84	N<br>Mean (SD)<br>Median<br>(IQR)	72<br>67.3 (170.3)<br>23.8 (13.8-56.2)<br>4-1399	24<br>58.3 (65.6)<br>32.2 (16.2-71.5)<br>9.7-268.1	24<br>98 (282.9)<br>21.8 (11.8-55)<br>5.4-1399	24<br>45.5 (60.3)<br>23.8 (13.1-52.5)<br>4-263	0.544
Day 112	N<br>Mean (SD)<br>Median<br>(IQR)	69<br>53 (108.8)<br>20.9 (13.5-44.1)<br>3.6-814	24<br>45.6 (57.7)<br>27.1 (14.3-46.3)<br>3.6-266.7	23<br>66 (169)<br>19.1 (9.8-33.6)<br>3.8-814	22<br>47.5 (67)<br>20.8 (16.5-44.7)<br>5.4-305.2	0.786
**PTH (pg/mL)**						
Baseline	N<br>Mean (SD)<br>Median<br>(IQR)	73<br>36.4 (14)<br>33.4 (25.9-43.1)<br>11.5-79.3	24<br>37.2 (15.4)<br>32.8 (25.6-43.1)<br>14.9-74.4	25<br>33.1 (10.5)<br>32.8 (25.5-40.9)<br>11.5-59.2	24<br>39.1 (15.7)<br>36 (27.2-48)<br>12.8-79.3	0.319
Day 28	N<br>Mean (SD)<br>Median<br>(IQR)	73<br>31.7 (11.6)<br>30.8 (23.3-39.8)<br>8.3-67.8	24<br>31.6 (10.4)<br>33.1 (25.4-38.2)<br>13.5-56.8	25<br>30.5 (11.3)<br>29.4 (23.3-39.8)<br>8.3-51.5	24<br>32.9 (13.3)<br>31.8 (23.9-42.1)<br>13-67.8	0.772
Day 56	N<br>Mean (SD)<br>Median<br>(IQR)	73<br>32.4 (14.5)<br>28.5 (23.6-37.9)<br>7.6-72.2	24<br>31.3 (13.6)<br>27.9 (23.5-34.6)<br>15.4-62.4	25<br>32.2 (13.1)<br>32.1 (25-39.5)<br>7.6-62.1	24<br>33.7 (17)<br>26.9 (22.5-38.7)<br>13.7-72.2	0.84
Day 84	N<br>Mean (SD)<br>Median<br>(IQR)	72<br>31.6 (12.6)<br>30.1 (23.2-37.1)<br>7.2-78.6	24<br>32.6 (11.9)<br>28.4 (25.3-37.5)<br>14-60.9	24<br>28 (11.3)<br>27.5 (20.8-34.4)<br>7.2-56.9	24<br>34.1 (14.2)<br>31.3 (27.7-38.2)<br>13.3-78.6	0.221
Day 112*	N<br>Mean (SD)<br>Median<br>(IQR)	69<br>34 (13)<br>31.1 (25.7-42.1)<br>9.2-73.1	24<br>34.9 (12.5)<br>29.9 (26.7-45.5)<br>18.4-63.2	23<br>32.3 (9.9)<br>32.5 (27-36.3)<br>10.2-50	22<br>34.8 (16.4)<br>30.4 (23.9-41.7)<br>9.2-73.1	0.753
**Ionized calcium (mmol/L)**						
Baseline	N<br>Mean (SD)<br>Median<br>(IQR)	73<br>1.2 (0)<br>1.2 (1.2-1.2)<br>1.2-1.3	24<br>1.2 (0)<br>1.2 (1.2-1.3)<br>1.2-1.3	25<br>1.2 (0)<br>1.2 (1.2-1.2)<br>1.2-1.3	24<br>1.2 (0)<br>1.2 (1.2-1.2)<br>1.2-1.3	0.208
Day 28	N<br>Mean (SD)<br>Median	72<br>1.2 (0)	24<br>1.2 (0)	24<br>1.2 (0)	24<br>1.2 (0)	0.636

	(IQR)	1.2 (1.2-1.2)<br>1-1.3	1.2 (1.2-1.2)<br>1-1.3	1.2 (1.2-1.2)<br>1.2-1.3	1.2 (1.2-1.2)<br>1.2-1.3	
Day 56	N<br>Mean (SD)<br>Median<br>(IQR)	73<br>1.2 (0)<br>1.2 (1.2-1.3)<br>1.2-1.3	24<br>1.2 (0)<br>1.2 (1.2-1.3)<br>1.2-1.3	25<br>1.2 (0)<br>1.2 (1.2-1.3)<br>1.2-1.3	24<br>1.2 (0)<br>1.2 (1.2-1.3)<br>1.2-1.3	0.486
Day 84	N<br>Mean (SD)<br>Median<br>(IQR)	72<br>1.2 (0)<br>1.2 (1.2-1.2)<br>1.2-1.3	24<br>1.2 (0)<br>1.2 (1.2-1.2)<br>1.2-1.3	24<br>1.2 (0)<br>1.2 (1.2-1.2)<br>1.2-1.3	24<br>1.2 (0)<br>1.2 (1.2-1.2)<br>1.2-1.3	0.674
Day 112	N<br>Mean (SD)<br>Median<br>(IQR)	69<br>1.2 (0)<br>1.2 (1.2-1.2)<br>1.1-1.3	24<br>1.2 (0)<br>1.2 (1.2-1.2)<br>1.2-1.3	23<br>1.2 (0)<br>1.2 (1.2-1.2)<br>1.2-1.3	22<br>1.2 (0)<br>1.2 (1.2-1.2)<br>1.1-1.3	0.373
**FGF-23 (pmol/L)**						
Baseline	N<br>Mean (SD)<br>Median<br>(IQR)	73<br>1 (1.6)<br>0.5 (0.3-1)<br>0-10.8	24<br>0.5 (0.4)<br>0.4 (0.2-0.8)<br>0-1.6	25<br>1.3 (2.2)<br>0.6 (0.3-1.1)<br>0-10.8	24<br>1.1 (1.4)<br>0.6 (0.3-1.1)<br>0-5.4	0.192
Day 28	N<br>Mean (SD)<br>Median<br>(IQR)	73<br>1.4 (2.8)<br>0.8 (0.4-1.4)<br>0-22.5	24<br>0.7 (0.6)<br>0.6 (0.4-0.8)<br>0-2.7	25<br>2.2 (4.4)<br>1.2 (0.4-1.7)<br>0-22.5	24<br>1.4 (1.8)<br>0.6 (0.4-1.5)<br>0-7.8	0.19
Day 56	N<br>Mean (SD)<br>Median<br>(IQR)	73<br>1.3 (1.6)<br>0.7 (0.3-1.4)<br>0-9.5	24<br>0.8 (0.9)<br>0.6 (0.3-1)<br>0-3.9	25<br>1.7 (2.2)<br>0.8 (0.5-2)<br>0-9.5	24<br>1.2 (1.4)<br>0.7 (0.4-1.5)<br>0.2-6.7	0.154
Day 84	N<br>Mean (SD)<br>Median<br>(IQR)	72<br>1.2 (1.4)<br>0.7 (0.3-1.3)<br>0-6.1	24<br>0.8 (0.9)<br>0.4 (0.3-1)<br>0-3.7	24<br>1.3 (1.4)<br>1 (0.2-1.6)<br>0-5.9	24<br>1.4 (1.6)<br>0.7 (0.5-1.3)<br>0-6.1	0.208
Day 112	N<br>Mean (SD)<br>Median<br>(IQR)	69<br>1.3 (1.9)<br>0.8 (0.4-1.2)<br>0.1-12	24<br>0.7 (0.5)<br>0.6 (0.3-1)<br>0.1-2.1	23<br>1.7 (2.6)<br>1 (0.4-1.8)<br>0.2-12	22<br>1.6 (1.8)<br>0.8 (0.6-2)<br>0.1-7.8	0.155
**CTX-I (ng/mL)**						
Baseline	N<br>Mean (SD)<br>Median<br>(IQR)	73<br>0.4 (0.2)<br>0.3 (0.2-0.5)<br>0.1-0.9	24<br>0.4 (0.2)<br>0.4 (0.3-0.5)<br>0.1-0.9	25<br>0.3 (0.2)<br>0.3 (0.2-0.4)<br>0.1-0.8	24<br>0.3 (0.1)<br>0.4 (0.3-0.4)<br>0.1-0.5	0.022
Day 28	N<br>Mean (SD)<br>Median<br>(IQR)	73<br>0.4 (0.2)<br>0.4 (0.3-0.5)<br>0.1-1.1	24<br>0.4 (0.2)<br>0.4 (0.3-0.6)<br>0.1-1.1	25<br>0.3 (0.1)<br>0.3 (0.2-0.4)<br>0.1-0.7	24<br>0.4 (0.2)<br>0.4 (0.3-0.5)<br>0.1-0.7	0.07
Day 56	N<br>Mean (SD)<br>Median	73<br>0.3 (0.2)<br>0.3 (0.2-0.5)<br>0-0.7	24<br>0.4 (0.2)<br>0.4 (0.2-0.6)<br>0.1-0.7	25<br>0.3 (0.1)<br>0.2 (0.2-0.4)<br>0.1-0.6	24<br>0.3 (0.1)<br>0.3 (0.2-0.4)<br>0-0.5	0.042

	(IQR)					
Day 84	N<br>Mean (SD)<br>Median<br>(IQR)	72<br>0.4 (0.2)<br>0.3 (0.2-0.5)<br>0-0.8	24<br>0.4 (0.2)<br>0.4 (0.3-0.5)<br>0.2-0.8	24<br>0.3 (0.2)<br>0.3 (0.2-0.4)<br>0.1-0.8	24<br>0.3 (0.2)<br>0.3 (0.2-0.5)<br>0-0.7	0.068
Day 112	N<br>Mean (SD)<br>Median<br>(IQR)	69<br>0.3 (0.2)<br>0.3 (0.2-0.4)<br>0-0.8	24<br>0.4 (0.2)<br>0.4 (0.2-0.5)<br>0.1-0.8	23<br>0.3 (0.2)<br>0.3 (0.2-0.4)<br>0.1-0.6	22<br>0.3 (0.2)<br>0.3 (0.2-0.4)<br>0-0.6	0.09
**P1NP (ng/mL)**						
Baseline	N<br>Mean (SD)<br>Median<br>(IQR)	73<br>62.6 (24.1)<br>58 (45.9-72.2)<br>22.7-149.8	24<br>75.9 (29)<br>68.2 (57.9-90.9)<br>22.7-149.8	25<br>53.8 (18.7)<br>50.5 (42.5-57.1)<br>28.6-128.1	24<br>58.6 (18.2)<br>58.3 (43.5-71.4)<br>32.9-88.9	0.003
Day 28	N<br>Mean (SD)<br>Median<br>(IQR)	73<br>64.8 (25.4)<br>60.4 (49.6-70)<br>25.1-153.6	24<br>80.5 (33.7)<br>67.3 (57.7-102.9)<br>25.1-153.6	25<br>55.9 (15.5)<br>54.4 (47.9-61.3)<br>28.6-103.3	24<br>58.4 (15.4)<br>60.1 (46-68.7)<br>32.8-92.5	0.001
Day 56	N<br>Mean (SD)<br>Median<br>(IQR)	73<br>61.8 (22.4)<br>56.4 (46.5-75.4)<br>23.5-145.9	24<br>71.5 (28)<br>61.9 (53.5-82.2)<br>23.5-145.9	25<br>54.2 (16.3)<br>49.8 (45.3-61.8)<br>32.6-104.6	24<br>60.1 (18.8)<br>56.5 (44.1-75.6)<br>29.5-99.2	0.021
Day 84	N<br>Mean (SD)<br>Median<br>(IQR)	72<br>60.3 (25.7)<br>56.4 (41.9-70.7)<br>25.3-195.7	24<br>72.6 (34.2)<br>66.2 (53.1-87.3)<br>27.3-195.7	24<br>50.8 (18)<br>46.1 (37.8-63.1)<br>25.3-87.6	24<br>57.4 (16.7)<br>55.9 (42.3-67.3)<br>33.9-88.2	0.009
Day 112	N<br>Mean (SD)<br>Median<br>(IQR)	69<br>57.3 (20.7)<br>55.9 (42-66.7)<br>23.6-109.5	24<br>67.2 (23.2)<br>61.8 (53.7-81.9)<br>23.6-107.4	23<br>50.4 (16.1)<br>48.4 (41-55.2)<br>27.8-100	22<br>53.8 (18.8)<br>51.7 (37.9-65.5)<br>29.5-109.5	0.012
**BALP (ug/L)**						
Baseline	N<br>Mean (SD)<br>Median<br>(IQR)	73<br>15.6 (6.7)<br>13.9 (11.1-18.3)<br>5.8-40.7	24<br>18 (8.5)<br>16.4 (12.5-20.8)<br>7.3-40.7	25<br>13.8 (3.9)<br>12.8 (11.1-15.6)<br>7.5-24.7	24<br>15.2 (6.6)<br>14.2 (10.4-18.3)<br>5.8-33.5	0.08
Day 28	N<br>Mean (SD)<br>Median<br>(IQR)	73<br>15.1 (5.9)<br>14.2 (11.2-17.4)<br>3.3-34.5	24<br>17.2 (6.2)<br>16.6 (12.6-20.8)<br>6.7-33.2	25<br>13.7 (4.1)<br>12.9 (10.8-15.7)<br>8.7-24.2	24<br>14.5 (6.7)<br>14 (10.3-16.5)<br>3.3-34.5	0.093
Day 56	N<br>Mean (SD)<br>Median<br>(IQR)	73<br>14.8 (6.7)<br>13.6 (10.8-16.8)<br>5.6-44.6	24<br>17.1 (8.1)<br>15.3 (12.3-18.5)<br>6-44.6	25<br>13.1 (4)<br>12.7 (10.1-14.8)<br>6.3-20.7	24<br>14.5 (7)<br>13.4 (10.6-15.8)<br>5.6-33.8	0.105
Day 84	N<br>Mean (SD)<br>Median	72<br>14.5 (6.4)<br>13.2 (10.6-15.9)<br>5.7-37.6	24<br>17.2 (7.6)<br>15.3 (12.2-22)<br>5.7-37.6	24<br>12.3 (3.6)<br>11.3 (10.3-14.4)<br>7.3-21.1	24<br>14 (6.5)<br>13.3 (11-14.2)<br>6.2-36.5	0.027

	(IQR)					
Day 112	N<br>Mean (SD)<br>Median<br>(IQR)	69<br>13.8 (6.2)<br>12.4 (10.1-16.8)<br>4.2-43	24<br>16.1 (8.1)<br>13.8 (10.3-19.5)<br>6-43	23<br>12.1 (3.5)<br>11.2 (9.4-13.9)<br>7-20.9	22<br>13.1 (5.4)<br>12.4 (10-15.5)<br>4.2-27.9	0.065
**Dkk-1 (pmol/L)**						
Baseline	N<br>Mean (SD)<br>Median<br>(IQR)	73<br>18.6 (11.1)<br>15.7 (10.6-23.1)<br>2.2-56	24<br>16.7 (8.3)<br>17.3 (11.9-23.2)<br>2.2-32.6	25<br>17.7 (12.6)<br>13.7 (10.3-21)<br>4-50.3	24<br>21.2 (11.9)<br>18.6 (13.3-26.4)<br>6.1-56	0.339
Day 28	N<br>Mean (SD)<br>Median<br>(IQR)	73<br>19.1 (11.2)<br>16 (10.7-23.6)<br>4-60.8	24<br>18 (8.6)<br>16 (12.2-23.4)<br>5.6-39.9	25<br>17.5 (11)<br>15.3 (9.7-20.6)<br>4-44.4	24<br>21.9 (13.3)<br>18.1 (12.5-27.7)<br>7-60.8	0.329
Day 56	N<br>Mean (SD)<br>Median<br>(IQR)	73<br>22.7 (12.1)<br>21.1 (13.8-31.6)<br>5-64.5	24<br>21.3 (10.1)<br>21.6 (12.6-27.5)<br>5-37.5	25<br>21.5 (12.1)<br>16.6 (11.4-31.6)<br>5.6-45.5	24<br>25.4 (13.8)<br>22.4 (14.7-32.4)<br>8.7-64.5	0.421
Day 84	N<br>Mean (SD)<br>Median<br>(IQR)	72<br>24.8 (14.2)<br>21.7 (12.7-34.5)<br>4.5-63.6	24<br>23.7 (13.1)<br>20.2 (13.7-31.8)<br>6-53	24<br>24.7 (14.3)<br>22.3 (11.9-35)<br>6.3-53.3	24<br>25.9 (15.5)<br>22.6 (14.3-35.4)<br>4.5-63.6	0.873
Day 112	N<br>Mean (SD)<br>Median<br>(IQR)	69<br>23.9 (12.7)<br>21.6 (14.1-31.4)<br>5-54.5	24<br>21.8 (11.7)<br>21.4 (11.8-30.1)<br>5.3-45.4	23<br>23.3 (13.2)<br>21.6 (13-30.2)<br>5-51.9	22<br>26.7 (13.1)<br>22.1 (18.2-33.1)<br>9.9-54.5	0.419
**Sclerostin (pmol/L)**						
Baseline	N<br>Mean (SD)<br>Median<br>(IQR)	73<br>25 (25.5)<br>18.9 (11.9-31.7)<br>4.1-173	24<br>20.5 (13.7)<br>16.8 (11.8-24.4)<br>4.1-66.2	25<br>28.2 (27)<br>19.5 (11.9-36.4)<br>5.8-139.1	24<br>26.1 (32.5)<br>19.2 (15.5-25.1)<br>5-173	0.559
Day 28	N<br>Mean (SD)<br>Median<br>(IQR)	73<br>22.3 (20.6)<br>17.9 (11.6-25.7)<br>0-151.8	24<br>18.1 (13.1)<br>16.6 (9.1-22.9)<br>0-57.9	25<br>22.7 (15.9)<br>17.9 (13.8-28.6)<br>0-82.2	24<br>26 (29.3)<br>19.2 (14.7-25.5)<br>0-151.8	0.419
Day 56	N<br>Mean (SD)<br>Median<br>(IQR)	73<br>25.2 (22)<br>20.5 (14.2-31.7)<br>0-150	24<br>20.1 (15.2)<br>19.7 (11.1-25.9)<br>0-63.3	25<br>27.3 (20.6)<br>24.9 (15-33.9)<br>6.2-106.8	24<br>28.1 (28.2)<br>20 (15.9-32.7)<br>7.8-150	0.382
Day 84	N<br>Mean (SD)<br>Median<br>(IQR)	72<br>24 (17.9)<br>19 (13.3-28.3)<br>0-111.1	24<br>20.9 (13.2)<br>18.4 (11.2-26.7)<br>4.5-52.5	24<br>27 (18.7)<br>20.9 (14.9-34.8)<br>5.2-89.9	24<br>24.1 (21.1)<br>19.7 (13.2-25.5)<br>0-111.1	0.504
Day 112	N<br>Mean (SD)<br>Median	69<br>25.6 (16.8)<br>20.3 (14-33.4)<br>6.2-98	24<br>24.1 (13.9)<br>19 (16.4-32.5)<br>7.6-57.7	23<br>28 (20.7)<br>19.7 (13.8-33.9)<br>6.2-98	22<br>24.5 (15.6)<br>22 (13.6-28.7)<br>8.1-76	0.691

	(IQR)					

Supplementary Material Table 3. Percentage change of the tested biochemical parameters with respect to baseline at the various time points.

**Time**		**ALL subjects (n=73)**	**Group A (n=24)**	**Group B (n=25)**	**Group C (n=24)**		**ANOVA Results**
**1,25-dihydroxyvitamin D**							
Day 28 percentage change with respect to Baseline	N<br>Mean (SD)<br>95%CI<br>p-value#<br>Median (IQR)	73<br>34 (35)<br>25.8;42.2<br><0.001<br>33 (11.4;49.8)	24<br>50 (38.3)<br>33.8;66.1<br><0.001<br>40.7 (25.4;76.1)	25<br>25.8 (29.5)<br>13.6;38<br><0.001<br>28.6 (6.4;44.3)	24<br>26.6 (32.7)<br>12.7;40.4<br>0.001<br>19.5 (8.9;41)	P anova<br>P AvsB<br>P AvsC<br>P BvsC<br>P AvsB (Holm)<br>P AvsC (Holm)<br>P BvsC (Holm)	0.022<br>0.014<br>0.019<br>0.941<br>0.043<br>0.043<br>0.941
Day 56 percentage change with respect to Baseline	N<br>Mean (SD)<br>95%CI<br>p-value#<br>Median (IQR)	73<br>34.1 (30.8)<br>26.9;41.2<br><0.001<br>35.1 (11.6;49)	24<br>45.3 (29.1)<br>33;57.6<br><0.001<br>41.5 (26.3;51.4)	25<br>25.2 (29.8)<br>12.9;37.5<br><0.001<br>23.1 (0;42.1)	24<br>32.1 (31.2)<br>18.9;45.3<br><0.001<br>26 (10.5;51)	P anova<br>P AvsB<br>P AvsC<br>P BvsC<br>P AvsB (Holm)<br>P AvsC (Holm)<br>P BvsC (Holm)	0.067<br>0.022<br>0.133<br>0.425<br>0.067<br>0.267<br>0.425
Day 84 percentage change with respect to Baseline	N<br>Mean (SD)<br>95%CI<br>p-value#<br>Median (IQR)	72<br>31.5 (29.5)<br>24.6;38.4<br><0.001<br>30.9 (7.4;44.2)	24<br>34.2 (22.6)<br>24.7;43.8<br><0.001<br>32.7 (18.6;42.9)	24<br>23.7 (29)<br>11.4;35.9<br>0.001<br>22 (2.8;38.7)	24<br>36.6 (35)<br>21.8;51.4<br><0.001<br>32.3 (13.3;63.6)	P anova<br>P AvsB<br>P AvsC<br>P BvsC<br>P AvsB (Holm)<br>P AvsC (Holm)<br>P BvsC (Holm)	0.273<br>0.218<br>0.776<br>0.131<br>0.435<br>0.776<br>0.392
Day 112 percentage change with respect to Baseline	N<br>Mean (SD)<br>95%CI<br>p-value#<br>Median (IQR)	69<br>13.1 (26.2)<br>6.8;19.4<br><0.001<br>10 (-6.4;27.9)	24<br>14.4 (26.4)<br>3.2;25.5<br>0.014<br>11.2 (3.2;22)	23<br>11.1 (28.9)<br>-1.5;23.6<br>0.08<br>3.3 (-8.8;31.6)	22<br>13.9 (24.2)<br>3.2;24.7<br>0.013<br>8.1 (-6.1;29.5)	P anova<br>P AvsB<br>P AvsC<br>P BvsC<br>P AvsB (Holm)<br>P AvsC (Holm)<br>P BvsC (Holm)	0.9<br>0.672<br>0.959<br>0.717<br>1<br>1<br>1
**24,25-dihydroxyvitamin D**							
Day 28 percentage change with respect to Baseline	N<br>Mean (SD)<br>95%CI<br>p-value#<br>Median (IQR)	73<br>11.6 (134.8)<br>-19.9;43.1<br>0.464<br>-17.7 (-49.5;27.2)	24<br>-4.7 (49.5)<br>-25.6;16.2<br>0.645<br>-11.3 (-<br>33.7;27.7)	25<br>46.6 (213.1)<br>-41.4;134.5<br>0.285<br>-9.6 (-50.8;65.7)<br>-	24<br>-8.5 (70.8)<br>-38.4;21.4<br>0.562<br>-25.1 (-49.9;15.1)	P anova<br>P AvsB<br>P AvsC<br>P BvsC<br>P AvsB (Holm)<br>P AvsC (Holm)<br>P BvsC (Holm)	0.281<br>0.186<br>0.922<br>0.156<br>0.467<br>0.922<br>0.467

Day 56 percentage change with respect to Baseline	N<br>Mean (SD)<br>95%CI<br>p-value#<br>Median (IQR)	73<br>-0.8 (83)<br>-20.2;18.6<br>0.935<br>-25.3 (-57.7;29.5)	24<br>13.9 (93)<br>-25.3;53.2<br>0.47<br>-23.6 (-46.3;41.5)<br>-	25<br>9.2 (93.7)<br>-29.5;47.8<br>0.629<br>-13.2 (-53.4;47.8)	24<br>-25.9 (52.5)<br>-48.1;-3.7<br>0.024<br>-37.4 (-61.8;4.1)	P anova<br>P AvsB<br>P AvsC<br>P BvsC<br>P AvsB (Holm)<br>P AvsC (Holm)<br>P BvsC (Holm)	0.192<br>0.839<br>0.098<br>0.14<br>0.839<br>0.293<br>0.293
Day 84 percentage change with respect to Baseline	N<br>Mean (SD)<br>95%CI<br>p-value#<br>Median (IQR)	72<br>1.4 (91)<br>-20;22.8<br>0.898<br>-17.6 (-63.8;29.7)	24<br>15.6 (116)<br>-33.4;64.6<br>0.517<br>2.7 (-62.3;29.7)	24<br>-0.1 (68.1)<br>-28.9;28.7<br>0.995<br>-11.3 (-52.8;40.5)	24<br>-11.4 (84.1)<br>-46.9;24.1<br>0.515<br>-32.4 (-66.1;18.3)	P anova<br>P AvsB<br>P AvsC<br>P BvsC<br>P AvsB (Holm)<br>P AvsC (Holm)<br>P BvsC (Holm)	0.595<br>0.555<br>0.312<br>0.671<br>1<br>0.935<br>1
Day 112 percentage change with respect to Baseline	N<br>Mean (SD)<br>95%CI<br>p-value#<br>Median (IQR)	69<br>-1.2 (112.1)<br>-28.1;25.7<br>0.929<br>-29.3 (-61.9;21.1)	24<br>-9.9 (73.9)<br>-41.1;21.3<br>0.519<br>-27.3 (-69.7;17.9)	23<br>26.1 (172.1)<br>-48.4;100.5<br>0.475<br>-42.6 (-57.9;54.8)	22<br>-20.2 (49.2)<br>-42.1;1.6<br>0.067<br>-25.1 (-60.5;11.8)	P anova<br>P AvsB<br>P AvsC<br>P BvsC<br>P AvsB (Holm)<br>P AvsC (Holm)<br>P BvsC (Holm)	0.348<br>0.275<br>0.755<br>0.17<br>0.551<br>0.755<br>0.511
**PTH**							
Day 28 percentage change with respect to Baseline	N<br>Mean (SD)<br>95%CI<br>p-value#<br>Median (IQR)	73<br>-7.7 (35.2)<br>-15.9;0.5<br>0.065<br>-14.4 (-34.2;9.4)	24<br>-6.6 (40.8)<br>-23.8;10.7<br>0.438<br>-13.7 (-35.3;11.5)	25<br>-5 (33.9)<br>-19;9.1<br>0.473<br>-12.3 (-27.2;15.9)	24<br>-11.8 (31.2)<br>-24.9;1.4<br>0.078<br>-20 (-32.4;0)	P anova<br>P AvsB<br>P AvsC<br>P BvsC<br>P AvsB (Holm)<br>P AvsC (Holm)<br>P BvsC (Holm)	0.785<br>0.873<br>0.615<br>0.505<br>1<br>1<br>1
Day 56 percentage change with respect to Baseline	N<br>Mean (SD)<br>95%CI<br>p-value#<br>Median (IQR)	73<br>-7.1 (35.9)<br>-15.5;1.3<br>0.097<br>-15.2 (-27.6;6.6)	24<br>-9.4 (41.5)<br>-26.9;8.1<br>0.279<br>-20.4 (-29.1;3.2)	25<br>-0.1 (37.8)<br>-15.7;15.6<br>0.994<br>-7.9 (-21.4;7.6)	24<br>-12 (27.2)<br>-23.5;-0.6<br>0.04<br>-15.5 (-34.3;3.3)	P anova<br>P AvsB<br>P AvsC<br>P BvsC<br>P AvsB (Holm)<br>P AvsC (Holm)<br>P BvsC (Holm)	0.476<br>0.368<br>0.799<br>0.249<br>0.746<br>0.799<br>0.746
Day 84 percentage change with respect to Baseline	N<br>Mean (SD)<br>95%CI<br>p-value#<br>Median (IQR)	72<br>-8.3 (31.2)<br>-15.6;-1<br>0.026<br>-10.5 (-38;14.9)	24<br>-6.5 (28.5)<br>-18.5;5.5<br>0.275<br>-8.6 (-25.4;12.2)	24<br>-11.9 (31.6)<br>-25.2;1.4<br>0.078<br>-10.7 (-38;8)	24<br>-6.6 (34.2)<br>-21;7.9<br>0.356<br>-10.5 (-40.1;17.2)	P anova<br>P AvsB<br>P AvsC<br>P BvsC<br>P AvsB (Holm)<br>P AvsC (Holm)<br>P BvsC (Holm)	0.793<br>0.554<br>0.994<br>0.559<br>1<br>1<br>1
Day 112 percentage change with respect to Baseline	N<br>Mean (SD)<br>95%CI<br>p-value#<br>Median (IQR)	69<br>1.2 (38.8)<br>-8.2;10.5<br>0.804<br>-6.4 (-23.7;15.5)	24<br>3.9 (44.5)<br>-14.9;22.7<br>0.673<br>-3 (-27.3;21.8)	23<br>0.9 (25.3)<br>-10.1;11.8<br>0.87<br>-11.2 (-21.6;17.1)	22<br>-1.5 (44.9)<br>-21.4;18.4<br>0.877<br>-6.6 (-23.2;11.3)	P anova<br>P AvsB<br>P AvsC<br>P BvsC<br>P AvsB (Holm)<br>P AvsC (Holm)<br>P BvsC (Holm)	0.897<br>0.794<br>0.644<br>0.84<br>1<br>1<br>1
**Ionized calcium**							
Day 28 percentage change with respect to Baseline	N<br>Mean (SD)<br>95%CI<br>p-value#<br>Median (IQR)	72<br>-0.7 (3.2)<br>-1.5;0<br>0.048<br>-0.8 (-2.4;0.8)	24<br>-1.7 (3.8)<br>-3.3;-0.1<br>0.036<br>-1.2 (-3.2;0)	24<br>-0.2 (3.2)<br>-1.5;1.2<br>0.793<br>-0.4 (-2.6;1.1)	24<br>-0.4 (2.3)<br>-1.3;0.6<br>0.433<br>-0.8 (-1.7;0.8)	P anova<br>P AvsB<br>P AvsC<br>P BvsC<br>P AvsB (Holm)<br>P AvsC (Holm)<br>P BvsC (Holm)	0.189<br>0.094<br>0.144<br>0.825<br>0.281<br>0.287<br>0.825

Day 56 percentage change with respect to Baseline	N<br>Mean (SD)<br>95%CI<br>p-value#<br>Median (IQR)	73<br>0.8 (2.8)<br>0.1;1.4<br>0.022<br>0.9 (-1.6;2.5)	24<br>0.4 (2.9)<br>-0.8;1.6<br>0.51<br>0.4 (-1.2;2.4)<br>-5.6;5.9	25<br>0.8 (2.8)<br>-0.3;2<br>0.14<br>0.8 (-0.8;2.4)<br>-4;8.6	24<br>1.1 (2.8)<br>-0.1;2.2<br>0.08<br>2.4 (-1.6;3.3)<br>-3.2;6	P anova<br>P AvsB<br>P AvsC<br>P BvsC<br>P AvsB (Holm)<br>P AvsC (Holm)<br>P BvsC (Holm)	0.71<br>0.573<br>0.421<br>0.802<br>1<br>1<br>1
Day 84 percentage change with respect to Baseline	N<br>Mean (SD)<br>95%CI<br>p-value#<br>Median (IQR)	72<br>-0.1 (2.6)<br>-0.7;0.5<br>0.717<br>-0.4 (-1.6;1.7)	24<br>-0.4 (2.1)<br>-1.3;0.5<br>0.335<br>-0.8 (-1.6;1.6)	24<br>0.5 (2.7)<br>-0.6;1.7<br>0.363<br>0 (-0.8;1.9)	24<br>-0.4 (2.9)<br>-1.7;0.8<br>0.479<br>-0.8 (-1.8;0.4)	P anova<br>P AvsB<br>P AvsC<br>P BvsC<br>P AvsB (Holm)<br>P AvsC (Holm)<br>P BvsC (Holm)	0.359<br>0.22<br>0.983<br>0.212<br>0.635<br>0.983<br>0.635
Day 112 percentage change with respect to Baseline	N<br>Mean (SD)<br>95%CI<br>p-value#<br>Median (IQR)	69<br>-0.9 (2.5)<br>-1.5;-0.3<br>0.005<br>-0.8 (-2.5;0.8)	24<br>-1.2 (2.5)<br>-2.2;-0.1<br>0.032<br>-1.2 (-3.1;0.8)	23<br>-0.7 (2.3)<br>-1.7;0.3<br>0.152<br>-0.8 (-2.4;0.8)	22<br>-0.7 (2.7)<br>-1.9;0.5<br>0.234<br>0 (-2.5;0.6)	P anova<br>P AvsB<br>P AvsC<br>P BvsC<br>P AvsB (Holm)<br>P AvsC (Holm)<br>P BvsC (Holm)	0.775<br>0.533<br>0.546<br>0.99<br>1<br>1<br>1
**FGF-23**							
Day 28 percentage change with respect to Baseline	N<br>Mean (SD)<br>95%CI<br>p-value#<br>Median (IQR)	69<br>104.3 (241.4)<br>46.3;162.3<br>0.001<br>40.6 (-6.5;87.4)	23<br>93.1 (190.6)<br>10.7;175.5<br>0.029<br>26.7 (-31.9;118.2)	24<br>91.4 (174.9)<br>17.5;165.2<br>0.018<br>59.1 (10.1;92.5)	22<br>130.1 (340.7)<br>-20.9;281.2<br>0.088<br>34.3 (-5.6;73.2)	P anova<br>P AvsB<br>P AvsC<br>P BvsC<br>P AvsB (Holm)<br>P AvsC (Holm)<br>P BvsC (Holm)	0.835<br>0.981<br>0.613<br>0.592<br>1<br>1<br>1
Day 56 percentage change with respect to Baseline	N<br>Mean (SD)<br>95%CI<br>p-value#<br>Median (IQR)	69<br>121.5 (322.4)<br>44;198.9<br>0.003<br>27.3 (-30.8;142.9)	23<br>140.4 (289.1)<br>15.4;265.5<br>0.029<br>44.2 (-33.3;198.8)	24<br>74.9 (140.6)<br>15.5;134.3<br>0.016<br>17.9 (-16.8;123.1)	22<br>152.5 (472.8)<br>-57.2;362.1<br>0.145<br>30.4 (-24.7;109.4)	P anova<br>P AvsB<br>P AvsC<br>P BvsC<br>P AvsB (Holm)<br>P AvsC (Holm)<br>P BvsC (Holm)	0.682<br>0.492<br>0.902<br>0.422<br>1<br>1<br>1
Day 84 percentage change with respect to Baseline	N<br>Mean (SD)<br>95%CI<br>p-value#<br>Median (IQR)	68<br>90.6 (238.2)<br>32.9;148.3<br>0.003<br>19.1 (-29.9;107.2)	23<br>156 (366.9)<br>-2.7;314.6<br>0.054<br>17.9 (-36.5;149.9)	23<br>49.5 (123.5)<br>-3.9;102.9<br>0.068<br>-1.7 (-42;100.2)	22<br>65.3 (129.6)<br>7.8;122.7<br>0.028<br>59.1 (-8.7;84.6)	P anova<br>P AvsB<br>P AvsC<br>P BvsC<br>P AvsB (Holm)<br>P AvsC (Holm)<br>P BvsC (Holm)	0.267<br>0.132<br>0.204<br>0.824<br>0.397<br>0.407<br>0.824
Day 112 percentage change with respect to Baseline	N<br>Mean (SD)<br>95%CI<br>p-value#<br>Median (IQR)	66<br>164 (554.5)<br>27.7;300.3<br>0.019<br>50 (-8.5;108.5)	23<br>114.8 (220.4)<br>19.4;210.1<br>0.021<br>58.8 (-10.8;185)	22<br>43.4 (72.5)<br>11.2;75.5<br>0.011<br>42.6 (0.4;64)	21<br>344.2 (942.1)<br>-84.6;773<br>0.11<br>50 (-21.9;137.7)	P anova<br>P AvsB<br>P AvsC<br>P BvsC<br>P AvsB (Holm)<br>P AvsC (Holm)<br>P BvsC (Holm)	0.18<br>0.664<br>0.17<br>0.077<br>0.664<br>0.341<br>0.23
**CTX-I**							
Day 28 percentage change with respect to Baseline	N<br>Mean (SD)<br>95%CI<br>p-value#<br>Median (IQR)	73<br>12.8 (38.1)<br>3.9;21.7<br>0.005<br>8.6 (-5.9;25)	24<br>8 (32.7)<br>-5.7;21.8<br>0.24<br>10.1 (-7.9;30)	25<br>16.3 (46.3)<br>-2.8;35.3<br>0.092<br>8.6 (-4.4;24.1)	24<br>14 (34.9)<br>-0.7;28.7<br>0.061<br>8 (-5.7;25.4)<br>-35;150	P anova<br>P AvsB<br>P AvsC<br>P BvsC<br>P AvsB (Holm)<br>P AvsC (Holm)<br>P BvsC (Holm)	0.746<br>0.458<br>0.594<br>0.839<br>1<br>1<br>1

Day 56 percentage change with respect to Baseline	N<br>Mean (SD)<br>95%CI<br>p-value#<br>Median (IQR)	73<br>1.6 (48.7)<br>-9.7;13<br>0.778<br>-5.6 (-27.5;16.7)	24<br>-3.5 (33.2)<br>-17.5;10.5<br>0.613<br>4.3 (-21.6;9.9)	25<br>9.5 (61.7)<br>-16;34.9<br>0.451<br>0 (-27.7;43.3)	24<br>-1.5 (47.4)<br>-21.5;18.6<br>0.881<br>-15.8 (-30.5;6.7)	P anova<br>P AvsB<br>P AvsC<br>P BvsC<br>P AvsB (Holm)<br>P AvsC (Holm)<br>P BvsC (Holm)	0.611<br>0.359<br>0.887<br>0.439<br>1<br>1<br>1
Day 84 percentage change with respect to Baseline	N<br>Mean (SD)<br>95%CI<br>p-value#<br>Median (IQR)	72<br>5.7 (40)<br>-3.7;15.1<br>0.229<br>0 (-20.1;25)	24<br>4 (28.4)<br>-8;16<br>0.5<br>4.5 (-6.7;23.9)	24<br>11.9 (54.5)<br>-11.1;35<br>0.294<br>-4.5 (-24.6;28.8)	24<br>1.3 (33.2)<br>-12.8;15.3<br>0.855<br>0 (-22.4;10.6)	P anova<br>P AvsB<br>P AvsC<br>P BvsC<br>P AvsB (Holm)<br>P AvsC (Holm)<br>P BvsC (Holm)	0.636<br>0.496<br>0.816<br>0.362<br>1<br>1<br>1
Day 112 percentage change with respect to Baseline	N<br>Mean (SD)<br>95%CI<br>p-value#<br>Median (IQR)	69<br>1.5 (53.1)<br>-11.3;14.2<br>0.817<br>-7.3 (-32.5;17.6)	24<br>-2.2 (34.4)<br>-16.7;12.3<br>0.759<br>-2.9 (-23.3;18)	23<br>9 (58.1)<br>-16.1;34.1<br>0.466<br>-2.6 (-32.4;23.2)	22<br>-2.4 (64.7)<br>-31.1;26.3<br>0.865<br>-17.6 (-38;0)	P anova<br>P AvsB<br>P AvsC<br>P BvsC<br>P AvsB (Holm)<br>P AvsC (Holm)<br>P BvsC (Holm)	0.714<br>0.477<br>0.99<br>0.479<br>1<br>1<br>1
**P1NP**							
Day 28 percentage change with respect to Baseline	N<br>Mean (SD)<br>95%CI<br>p-value#<br>Median (IQR)	73<br>4.9 (17.2)<br>0.9;8.9<br>0.018<br>3 (-8.3;15.2)	24<br>6.7 (20.2)<br>-1.9;15.2<br>0.12<br>3.8 (-2.6;14.6)	25<br>5.8 (15.2)<br>-0.5;12<br>0.069<br>3 (-5.5;16.5)	24<br>2.1 (16.1)<br>-4.7;8.9<br>0.531<br>2.6 (-10.8;9.5)	P anova<br>P AvsB<br>P AvsC<br>P BvsC<br>P AvsB (Holm)<br>P AvsC (Holm)<br>P BvsC (Holm)	0.624<br>0.855<br>0.362<br>0.459<br>1<br>1<br>1
Day 56 percentage change with respect to Baseline	N<br>Mean (SD)<br>95%CI<br>p-value#<br>Median (IQR)	73<br>1 (18.4)<br>-3.3;5.3<br>0.638<br>-1.1 (-11;14.5)	24<br>-3.6 (20.3)<br>-12.2;5<br>0.394<br>-7.8 (-15.7;8.5)	25<br>2.5 (15.6)<br>-4;8.9<br>0.439<br>0.8 (-9.3;15.2)	24<br>4.2 (19)<br>-3.9;12.2<br>0.296<br>7.1 (-8.2;15.4)	P anova<br>P AvsB<br>P AvsC<br>P BvsC<br>P AvsB (Holm)<br>P AvsC (Holm)<br>P BvsC (Holm)	0.313<br>0.253<br>0.148<br>0.747<br>0.506<br>0.445<br>0.747
Day 84 percentage change with respect to Baseline	N<br>Mean (SD)<br>95%CI<br>p-value#<br>Median (IQR)	72<br>-2.4 (20.5)<br>-7.2;2.4<br>0.324<br>-3.8 (-14.7;11.3)	24<br>-3 (21.8)<br>-12.2;6.2<br>0.506<br>-5.6 (-14.6;7.9)	24<br>-5.3 (19)<br>-13.3;2.8<br>0.187<br>-7.5 (-19.9;10.8)	24<br>1.1 (20.9)<br>-7.7;9.9<br>0.799<br>-0.5 (-8.4;21.1)	P anova<br>P AvsB<br>P AvsC<br>P BvsC<br>P AvsB (Holm)<br>P AvsC (Holm)<br>P BvsC (Holm)	0.556<br>0.703<br>0.493<br>0.287<br>0.985<br>0.985<br>0.86
Day 112 percentage change with respect to Baseline	N<br>Mean (SD)<br>95%CI<br>p-value#<br>Median (IQR)	69<br>-7.1 (18)<br>-11.4;-2.8<br>0.002<br>-8.6 (-17.6;3.5)	24<br>-9.2 (16)<br>-16;-2.5<br>0.01<br>-8.7 (-20.3;4.1)	23<br>-6.3 (14.1)<br>-12.3;-0.2<br>0.044<br>-8.6 (-14.4;3)	22<br>-5.6 (23.5)<br>-16;4.8<br>0.275<br>-8.8 (-17;3.5)	P anova<br>P AvsB<br>P AvsC<br>P BvsC<br>P AvsB (Holm)<br>P AvsC (Holm)<br>P BvsC (Holm)	0.769<br>0.576<br>0.503<br>0.908<br>1<br>1<br>1
**BALP**							
Day 28 percentage change with respect to Baseline	N<br>Mean (SD)<br>95%CI<br>p-value#<br>Median (IQR)	73<br>-1.8 (15.6)<br>-5.4;1.9<br>0.333<br>-1.7 (-10.7;7.9)	24<br>-0.4 (15.9)<br>-7.1;6.3<br>0.905<br>-1.3 (-8.2;7.6)	25<br>-0.1 (12.1)<br>-5.1;4.9<br>0.962<br>-2.8 (-8.9;8.1)	24<br>-4.9 (18.5)<br>-12.7;2.9<br>0.207<br>-6.7 (-15.4;5.3)	P anova<br>P AvsB<br>P AvsC<br>P BvsC<br>P AvsB (Holm)<br>P AvsC (Holm)<br>P BvsC (Holm)	0.494<br>0.951<br>0.321<br>0.288<br>0.951<br>0.865<br>0.865

Day 56 percentage change with respect to Baseline	N<br>Mean (SD)<br>95%CI<br>p-value#<br>Median (IQR)	73<br>-4.4 (14.5)<br>-7.8;-1<br>0.011<br>-2.4 (-15.4;5.2)	24<br>-3.5 (16.7)<br>-10.5;3.5<br>0.316<br>-7.7 (-15.4;9)	25<br>-5.1 (13.5)<br>-10.7;0.5<br>0.073<br>-2.4 (-8.9;3)	24<br>-4.7 (13.8)<br>-10.5;1.1<br>0.109<br>-0.2 (-14;5.2)	P anova<br>P AvsB<br>P AvsC<br>P BvsC<br>P AvsB (Holm)<br>P AvsC (Holm)<br>P BvsC (Holm)	0.926<br>0.707<br>0.779<br>0.927<br>1<br>1<br>1
Day 84 percentage change with respect to Baseline	N<br>Mean (SD)<br>95%CI<br>p-value#<br>Median (IQR)	72<br>-6.5 (16.7)<br>-10.4;-2.6<br>0.001<br>-6.8 (-17.9;5.5)	24<br>-2.5 (18.2)<br>-10.2;5.2<br>0.503<br>-2.2 (-16.1;9.9)	24<br>-10.1 (15.2)<br>-16.5;-3.6<br>0.004<br>-8.5 (-23.6;-3.6)	24<br>-6.9 (16.3)<br>-13.8;0<br>0.049<br>-11.1 (-17.1;5.5)	P anova<br>P AvsB<br>P AvsC<br>P BvsC<br>P AvsB (Holm)<br>P AvsC (Holm)<br>P BvsC (Holm)	0.296<br>0.122<br>0.365<br>0.514<br>0.365<br>0.731<br>0.731
Day 112 percentage change with respect to Baseline	N<br>Mean (SD)<br>95%CI<br>p-value#<br>Median (IQR)	69<br>-10.0 (16.4)<br>-14.5;-6.6<br><0.001<br>-10.6 (-22.4;-1.1)	24<br>-9.4 (15.9)<br>-16.1;-2.6<br>0.008<br>-8.3 (-21.8;-0.2)	23<br>-11.5 (17.5)<br>-19;-3.9<br>0.005<br>-13 (-22.8;-3.3)	22<br>-11 (16.5)<br>-18.3;-3.7<br>0.005<br>-10 (-20.6;-1.3)	P anova<br>P AvsB<br>P AvsC<br>P BvsC<br>P AvsB (Holm)<br>P AvsC (Holm)<br>P BvsC (Holm)	0.903<br>0.668<br>0.743<br>0.924<br>1<br>1<br>1
**Dkk-1**							
Day 28 percentage change with respect to Baseline	N<br>Mean (SD)<br>95%CI<br>p-value#<br>Median (IQR)	73<br>15.6 (66.9)<br>0;31.2<br>0.05<br>-0.3 (-16.3;20.7)	24<br>28.9 (97.3)<br>-12.2;70<br>0.159<br>9.1 (-8.1;42.4)	25<br>11 (48.7)<br>-9.2;31.1<br>0.272<br>-4.4 (-16.5;18.8)	24<br>7.1 (42.1)<br>-10.6;24.9<br>0.416<br>-4.5 (-22.3;21.2)	P anova<br>P AvsB<br>P AvsC<br>P BvsC<br>P AvsB (Holm)<br>P AvsC (Holm)<br>P BvsC (Holm)	0.489<br>0.352<br>0.265<br>0.842<br>0.794<br>0.794<br>0.842
Day 56 percentage change with respect to Baseline	N<br>Mean (SD)<br>95%CI<br>p-value#<br>Median (IQR)	73<br>41.2 (76.4)<br>23.4;59.1<br><0.001<br>21.7 (-3.3;60.8)	24<br>51.5 (104)<br>7.6;95.4<br>0.024<br>34.6 (1.4;53.8)	25<br>39.3 (59.3)<br>14.8;63.7<br>0.003<br>20.5 (-9.6;95.6)	24<br>33.1 (60)<br>7.7;58.4<br>0.013<br>14.9 (-1;58.5)	P anova<br>P AvsB<br>P AvsC<br>P BvsC<br>P AvsB (Holm)<br>P AvsC (Holm)<br>P BvsC (Holm)	0.703<br>0.582<br>0.411<br>0.779<br>1<br>1<br>1
Day 84 percentage change with respect to Baseline	N<br>Mean (SD)<br>95%CI<br>p-value#<br>Median (IQR)	72<br>48.1 (77.6)<br>29.9;66.3<br><0.001<br>18.5 (-2.5;80.7)	24<br>60.9 (94.3)<br>21.1;100.7<br>0.004<br>30.3 (2.9;81.2)	24<br>52.7 (73.2)<br>21.8;83.6<br>0.002<br>27.1 (0;86.7)	24<br>30.7 (62)<br>4.5;56.9<br>0.023<br>12.7 (-6.5;54.7)	P anova<br>P AvsB<br>P AvsC<br>P BvsC<br>P AvsB (Holm)<br>P AvsC (Holm)<br>P BvsC (Holm)	0.385<br>0.718<br>0.183<br>0.33<br>0.718<br>0.549<br>0.659
Day 112 percentage change with respect to Baseline	N<br>Mean (SD)<br>95%CI<br>p-value#<br>Median (IQR)	69<br>46.1 (73.5)<br>28.5;63.8<br><0.001<br>24.1 (-6;83.5)	24<br>51.2 (88)<br>14.1;88.4<br>0.009<br>17.8 (-5.3;93.8)	23<br>49.3 (72.2)<br>18.1;80.6<br>0.003<br>24.1 (0.3;78.2)	22<br>37.3 (58.6)<br>11.3;63.3<br>0.007<br>34.3 (-6.7;70.2)	P anova<br>P AvsB<br>P AvsC<br>P BvsC<br>P AvsB (Holm)<br>P AvsC (Holm)<br>P BvsC (Holm)	0.792<br>0.93<br>0.527<br>0.588<br>1<br>1<br>1
**Sclerostin**							
Day 28 percentage change with respect to Baseline	N<br>Mean (SD)<br>95%CI<br>p-value#<br>Median (IQR)	73<br>-2.0 (47.6)<br>-13.0;9.0<br>0.715<br>-7.5 (-35.9;35.4)	25<br>-5.1 (49.0)<br>-25.3;15.1<br>0.608<br>-17 (-41.0;31.8)	24<br>-2.5 (40.8)<br>-23.2;18.5<br>0.809<br>-7.6 (-42.0;33.0)	24<br>1.64 (44.4)<br>-17.0;20.4<br>0.59<br>6.5 (-25.7;46.3)	P anova<br>P AvsB<br>P AvsC<br>P BvsC<br>P AvsB (Holm)<br>P AvsC (Holm)<br>P BvsC (Holm)	0.849<br>0.694<br>0.873<br>0.58<br>1<br>1<br>1

Day 56 percentage change with respect to Baseline	N<br>Mean (SD)<br>95%CI<br>p-value#<br>Median (IQR)	73<br>8 (46.5)<br>-13.1;9.0<br>0.146<br>-7.65 (-37.7;37.0)	24<br>-6.0 (52.3)<br>-28.5;15.7<br>0.550<br>-6.0 (-42.1;45.0)	25<br>10.5 (35.47)<br>-4.1;25.2<br>0.150<br>2.1 (-13.4;35.3)	24<br>19.8 (48.7)<br>(-0.8;40.3)<br>0.059<br>8.52 (-19.7;67.4)	P anova<br>P AvsB<br>P AvsC<br>P BvsC<br>P AvsB (Holm)<br>P AvsC (Holm)<br>P BvsC (Holm)	0.426<br>0.872<br>0.231<br>0.294<br>0.872<br>0.693<br>0.693
Day 84 percentage change with respect to Baseline	N<br>Mean (SD)<br>95%CI<br>p-value#<br>Median (IQR)	72<br>4.7 (49.7)<br>-3.0;20.4<br>0.143<br>2.5 (-32.2;50.0)	24<br>-5.9 (49.4)<br>-14.9;26.8<br>0.560<br>5.27 (-36.1;49.4)	24<br>15.9 (9.8)<br>-4.27;36.2<br>0.116<br>9.9 (-29.4;52.0)	24<br>4.1 (53.1)<br>-18.3;26.5<br>0.706<br>-6.1 (-35.6;51.1)	P anova<br>P AvsB<br>P AvsC<br>P BvsC<br>P AvsB (Holm)<br>P AvsC (Holm)<br>P BvsC (Holm)	0.999<br>0.963<br>0.972<br>0.991<br>1<br>1<br>1
Day 112 percentage change with respect to Baseline	N<br>Mean (SD)<br>95%CI<br>p-value#<br>Median (IQR)	69<br>8.2 (35.8)<br>-0.02;16.4<br>0.051<br>10.8 (-21.2;33.3)	24<br>12.7 (36.2)<br>-2.21;27.7<br>0.091<br>17.9 (-21.2;50.0)	23<br>10.5 (38.1)<br>-5.2;26.2<br>0.18<br>5.8 (-13.8;31.6)	22<br>1.4 (33.4)<br>-12.4;15.2<br>0.836<br>5.9 (-29.7;30.0)	P anova<br>P AvsB<br>P AvsC<br>P BvsC<br>P AvsB (Holm)<br>P AvsC (Holm)<br>P BvsC (Holm)	0.404<br>0.212<br>0.86<br>0.294<br>0.635<br>0.86<br>0.635

BALB = bone alkaline phosphatase; CTX-1 = C-terminal telopeptide of type I collagen; DKK-1 = Dicckopf-1; FGF-23 = fibroblast growth factor-23; IQR = interquartile range; P1NP = procollagen type I N-terminal propeptide; PTH = parathyroid hormone; SD = standard deviation
The Holm–Bonferroni method was used to counteract the problem of multiple comparisons.
